# Solid‐State Electrochemical Thermal Switches with Large Thermal Conductivity Switching Widths

**DOI:** 10.1002/advs.202401331

**Published:** 2024-06-25

**Authors:** Zhiping Bian, Mitsuki Yoshimura, Ahrong Jeong, Haobo Li, Takashi Endo, Yasutaka Matsuo, Yusaku Magari, Hidekazu Tanaka, Hiromichi Ohta

**Affiliations:** ^1^ Graduate School of Information Science and Technology Hokkaido University N14W9, Kita Sapporo 060‐0814 Japan; ^2^ Research Institute for Electronic Science Hokkaido University N20W10, Kita Sapporo 001–0020 Japan; ^3^ SANKEN Osaka University Mihogaoka 8‐1 Ibaraki Osaka 567‐0047 Japan

**Keywords:** electrochemical redox reaction, thermal conductivity, thermal switches

## Abstract

Thermal switches that switch the thermal conductivity (*κ*) of the active layers are attracting increasing attention as thermal management devices. For electrochemical thermal switches, several transition metal oxides (TMOs) are proposed as active layers. After electrochemical redox treatment, the crystal structure of the TMO is modulated, which results in the *κ* switching. However, the *κ* switching width is still small (<4 W m^−1^ K^−1^). In this study, it demonstrates that LaNiO*_x_*‐based solid‐state electrochemical thermal switches have a *κ* switching width of 4.3 W m^−1^ K^−1^. Fully oxidized LaNiO_3_ (on state) has a *κ* of 6.0 W m^−1^ K^−1^ due to the large contribution of electron thermal conductivity (*κ*_ele_, 3.1 W m^−1^ K^−1^). In contrast, reduced LaNiO_2.72_ (off state) has a *κ* of 1.7 W m^−1^ K^−1^ because the phonons are scattered by the oxygen vacancies. The LaNiO*_x_*‐based electrochemical thermal switch is cyclable of *κ* and the crystalline lattice of LaNiO*_x_*. This electrochemical thermal switch may be a promising platform for next‐generation devices such as thermal displays.

## Graphical abstract

Solid‐state electrochemical thermal switches that exhibit a wide thermal conductivity switching width of 4.3 W m^−1^K^−1^ are demonstrated. The oxidized on‐state exhibited 6.0 W m^−1^K^−1^, due to a large contribution of electrons, whereas the reduced off‐state exhibited 1.7 W m^−1^K^−1^. The present results will boost the development of solid‐state electrochemical thermal switches for thermal management devices.

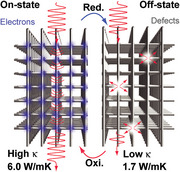

## 1 Introduction

Reuse of waste heat resulting from the low conversion rate of primary energy is crucial for sustainable development. Low‐ to medium‐temperature (100–300 °C) waste heat is the most difficult to reuse; the temperature is too low to generate jet steam for power generation. Although thermoelectric energy conversion technology is a solution, its efficiency is too low in this temperature range in the air.^[^ ^1^, ^2^, ^3^, ^4^ ^]^ Thermal management technologies^[^ ^5^ ^]^ such as thermal diodes^[^ ^6^, ^7^, ^8^ ^]^ and thermal switches (or sometimes they are called as thermal transistors)^[^ ^9^, ^10^, ^11^, ^12^, ^13^, ^14^, ^15^, ^16^ ^]^ have recently attracted attention. Thermal diodes rectify the heat flow; thermal switches electrically switch the heat flow on and off. We expect that thermal displays that visualize heat contrast using infrared cameras can be realized using thermal switches. Thus, thermal switches may be useful for the reuse of waste heat.

For this purpose, electrical control of thermal conductivity (*κ*) in the active materials of thermal switches is paramount. It necessitates a switch between the on‐state (high *κ*) and off‐state (low *κ*). Electrochemical^[^ ^10^, ^11^, ^12^, ^13^, ^14^ ^]^ and electrostatic^[^ ^15^, ^16^ ^]^ approaches offer pathways to govern the *κ* of active materials. Although electrostatic methods provide rapid *κ* control, their suitability for thermal display applications is limited by the requirement of an extremely thin active material around the heterointerface between the gate dielectric and the active material. We focus on electrochemical methods because they control the *κ* of entire materials. Many studies have used ionic liquids such as organic electrolytes and water for electrochemical modulation of materials.^[^ ^10^, ^11^, ^12^, ^14^ ^]^ However, this method is incompatible with integrated circuits, limiting its application. In our pursuit of thermal displays with substantial thermal conductance differences between the on‐ and off‐states, we used all‐solid‐state electrochemical thermal switches.^[^ ^13^, ^17^, ^18^ ^]^

All‐solid‐state electrochemical thermal switches, a cornerstone of advanced thermal management, harness the redox modulation of transition metal oxides (TMOs). Among many candidates,^[^ ^9^, ^10^, ^11^, ^12^, ^13^, ^14^, ^15^, ^16^ ^]^ TMOs have emerged as promising materials. When subjected to electrochemical redox treatment by inserting and extracting metal ions^[^ ^10^ ^]^ and oxide ions,^[^ ^12^, ^13^, ^14^ ^]^ TMOs undergo structural transformations leading to a switch in their *κ* width. Despite these advancements, the challenge lies in achieving a substantial *κ* switching width, a critical factor for practical application that is often limited (<4 W m^−1^ K^−1^).^[^ ^10^, ^12^, ^13^, ^14^, ^19^ ^]^

**Figure 1:**
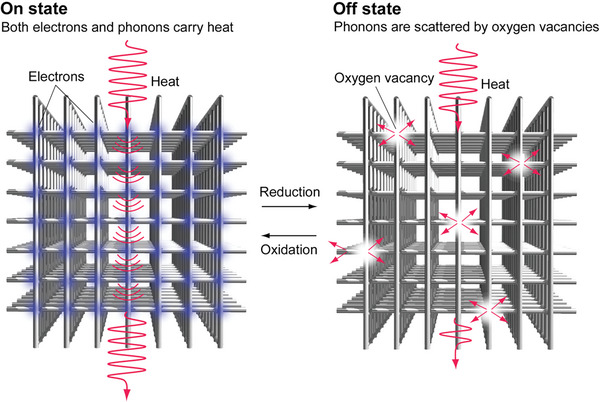
Strategy of electrochemical thermal switches with transition metal oxide active layer with large *κ* switching width. (Left) Diagram of on state. Fully oxidized TMOs show high electrical conductivity. Both electrons and phonons carry heat. The thermal switch shows high *κ*. (Right) Diagram of off state. Electrochemical reduction treatment produces oxygen vacancies. The reduced TMOs show low electrical conductivity. The *κ*_ele_ is negligible. Phonons are scattered by oxygen vacancies. The thermal switch shows low *κ*.

To overcome this limitation, we chose LaNiO_3_ as the active material for solid‐state electrochemical thermal switches. Bulk LaNiO_3_ has comparably high electrical and thermal conductivity,^[^ ^20^ ^]^ indicating its potential for *κ* modulation (Figure S1, Supporting Information). The schematic depicted in **Figure** **1** introduces LaNiO*_x_* as the active layer, offering a wide *κ* switching width. In the on state, LaNiO_3_ has heightened electrical conductivity when fully oxidized, resulting in a significant contribution from the electron thermal conductivity (*κ*_ele_). Conversely, the off state achieved through electrochemical reduction leads to oxygen vacancies, reduced electrical conductivity, and negligible *κ*_ele_. The scattering of phonons by these vacancies manifests as a low *κ*.

This study focuses on the use of LaNiO*_x_*‐based electrochemical thermal switches to address the limitations in *κ* switching width. Our investigations indicated a *κ* switching width of 4.3 W m^−1^ K^−1^. Fully oxidized LaNiO_3_ (on‐state) exhibited a high *κ* of 6.0 W m^−1^ K^−1^, primarily attributed to the contribution of *κ*_ele_ (3.1 W m^−1^ K^−1^). Conversely, reduced LaNiO_2.72_ (off state) had a low *κ* of 1.7 W m^−1^ K^−1^ due to the scattering of phonons by oxygen vacancies. Both the reduction and oxidation processes exhibited a nearly linear change in *κ*.

Furthermore, our study investigates the cyclability of *κ* and the crystalline lattice of LaNiO*_x_*‐based thermal switches. The performance of these electrochemical thermal switches positions them as viable candidates for integration into next‐generation devices, particularly thermal displays.

## 2 Results and Discussion

### 2.1 Electrochemical Thermal Switch Fabrication and Operation

**Figure 2:**
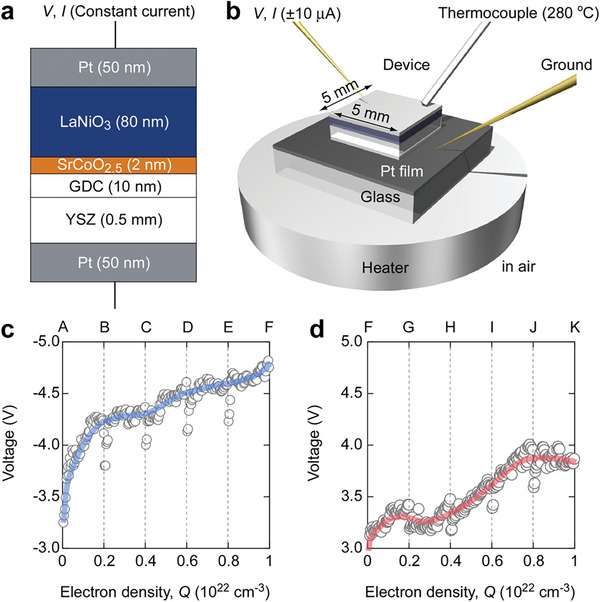
LaNiO*_x_*‐based thermal switch operation. a) Structure of the thermal switch that is composed of six layers: 50‐nm‐thick Pt film, 80‐nm‐thick LaNiO*_x_* film, 2‐nm‐thick SrCoO*_x_* film, 10‐nm‐thick Gd‐doped CeO_2_ (GDC) film, 0.5‐mm‐thick (001) YSZ single‐crystal substrate, and 40‐nm‐thick Pt film. b) Schematic of LaNiO*_x_* thermal switch. The switch was placed on a Pt‐coated glass substrate and heated at 280 °C in air. A K‐type thermocouple was used to monitor the switch surface temperature. The switch measured 5 mm × 5 mm. A constant negative current (−10 µA) was applied for reduction; a constant positive current (+10 µA) was applied for oxidation. c,d) Changes in observed DC voltage of thermal switch during reduction from LaNiO_3_ to LaNiO_2.72_ and oxidation from LaNiO_2.72_ to LaNiO_3_ with a step of *Q* = 2 × 10^21^ cm^−3^.

**Figure** **2**a shows a schematic of the thermal switch device structure, which is similar to those of our previous thermal switches.^[^ ^13^, ^17^, ^18^ ^]^ In this study, we inserted an extremely thin SrCoO*_x_* layer between the LaNiO_3_ and solid electrolyte (Gd‐doped CeO_2_/YSZ) (Figure S2, Supporting Information). As shown in Figure S2 (Supporting Information), when LaNiO_3_ was grown on the SrCoO*_x_*/GDC‐buffered (001) YSZ, the crystallographic orientation was stronger than that without a buffer layer. X‐ray reciprocal space mapping (RSM) around 113 YSZ diffraction spots (data not shown) confirmed that LaNiO_3_ grown on SrCoO*_x_*/GDC‐buffered (001) YSZ demonstrated the highest quality. Moreover, there was a positive correlation between the quality of LaNiO_3_ and its thermal conductivity (Table S3, Supporting Information). Thus, the use of the thermal switch structure shown in Figure 2a is crucial.

The setup for the operation of the LaNiO*_x_* thermal switch is shown in Figure 2b. Electrochemical redox treatment was performed at 280 °C in the air by applying a constant current of ±10 µA. During the redox reaction, we controlled the flown electron density *Q* = (*I·t*)/(*e·V*) through the current application time (Figure 2c,d), with a step of *Q* = 2 × 10^21^ cm^−3^ marked as A–K, where *I* is the flown current, *t* is the application time, *e* is the electron charge, and *V* is the volume of the LaNiO*_x_* layer in the thermal switch. In this study, electrochemical redox treatments were performed according to Faraday's law of electrolysis.

Reduction:

(1) $$\mathrm{LaNi}O_{3}+0.56e^{-}\to \mathrm{LaNi}O_{2.72}+0.28O^{2-}$$

Oxidation:

(2) $$\mathrm{LaNi}O_{2.72}+0.28O^{2-}\to \mathrm{LaNi}O_{3}+0.56e^{-}$$

The econductivity of the reduced state of LaNiO_2.72_ (9 S cm^−1^) also confirmed its identity as LaNiO_2.72_.^[^ ^21^ ^]^

Electrochemical redox treatment was initiated by applying a negative current to reduce LaNiO_3_ to LaNiO_2.72_ (Figure 2c). As *Q* increased, the absolute value of the voltage increased. The slope decreased after 2 × 10^21^ cm^−3^ and slightly increased after 2 × 10^21^ cm^−3^. When the current became positive, LaNiO_2.72_ was oxidized to LaNiO_3_ (Figure 2d). As *Q* increased, fluctuations occurred at 2 × 10^21^ cm^−3^ and 8 × 10^21^ cm^−3^. Overall, there was still an increasing trend and an absolute value between 3 and 5 V, with minimal variations. The absence of steps in the process curve indicates that there were no new thermodynamically stable phases.

### 2.2 Crystalline Lattice and *κ* Changes During Redox Treatment

**Figure 3:**
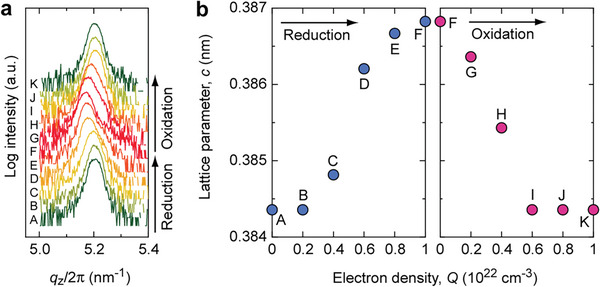
Change in the crystalline lattice of the LaNiO*_x_* layer after redox treatment. a) Change in out‐of‐plane XRD patterns after redox treatment with a step of *Q* = 2 × 10^21^ cm^−3^. The 002 diffraction peak shifted to a smaller *q*_z_ side after reduction and a larger *q*_z_ side after oxidation. These shifts were entirely reversible. b) Changes in lattice parameter *c* of LaNiO*_x_* as a function of *Q*. A lattice expansion of ≈0.6% occurred after reduction.

Redox treatment induced reversible changes in the crystalline lattice of LaNiO*_x_* step by step (reduction A → F, oxidation: F → K), as evidenced by the out‐of‐plane X‐ray diffraction patterns (**Figure** **3**a). Slight shifts in the 002 diffraction peak indicated modulations in the crystal structure, with lattice expansion observed after reduction and shrinkage observed after oxidation. The change in the lattice parameter *c* is shown in Figure 3b.

**Figure 4:**
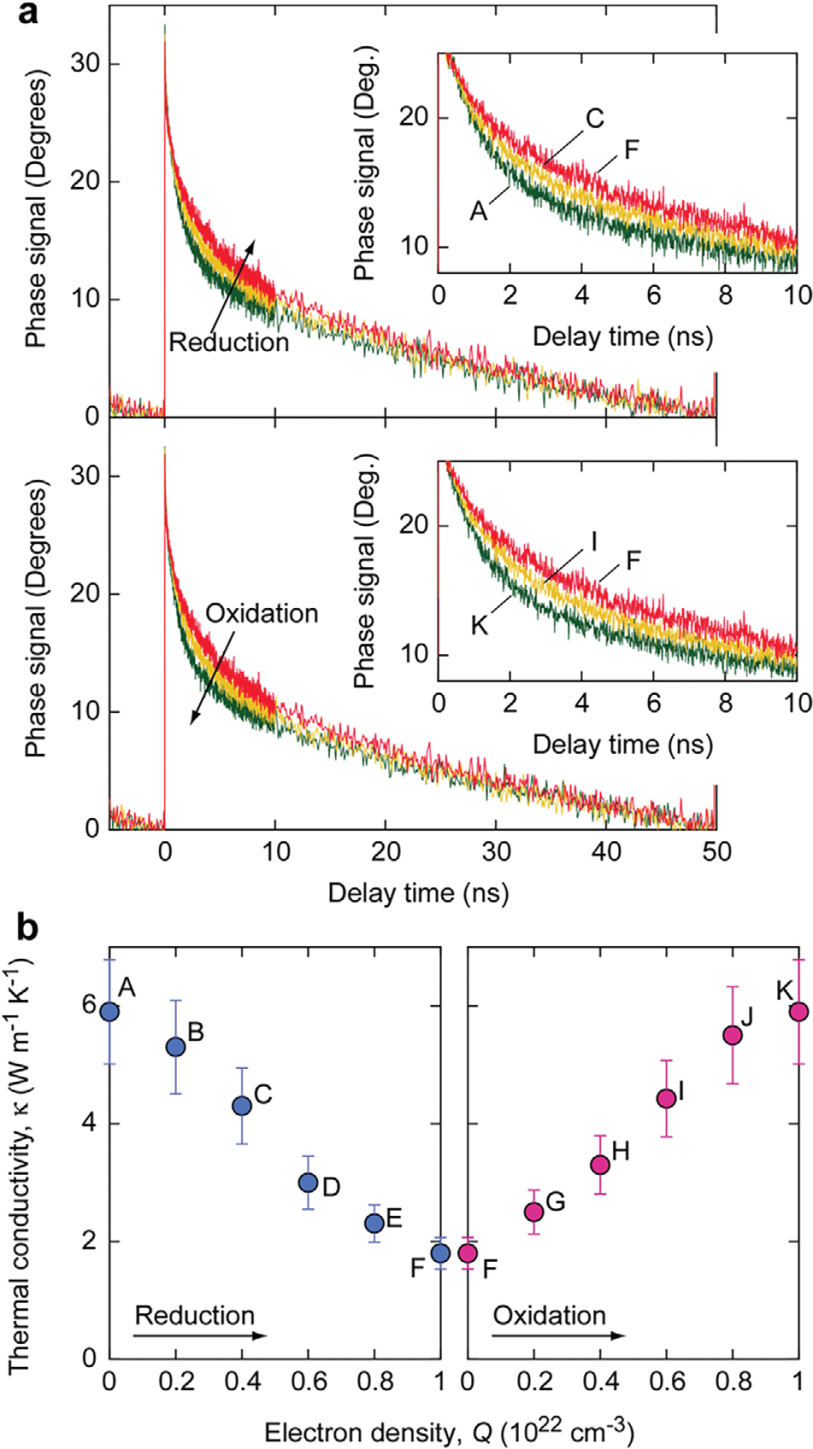
Change in *κ* of LaNiO*_x_* layer after redox treatment. a) TDTR decay curves of the thermal switch after (upper) reduction and (lower) oxidation treatment. b) Changes in *κ* of LaNiO*_x_* layer after (left) reduction and (right) oxidation treatment with a step of *Q* = 2 × 10^21^ cm^−3^. The *κ* of LaNiO_3_ was 5.9 W m^−1^ K^−1^; it decreased almost linearly with *Q* after reduction. After oxidation, the *κ* increased almost linearly with *Q* and returned to 5.9 W m^−1^ K^−1^.

**Figures** **4** and S3 (Supporting Information) illustrate the significant changes in the *κ* of the LaNiO*_x_* during redox treatment. Time‐domain thermoreflectance (TDTR) decay curves show a decrease in *κ* from 5.9 to 1.8 W m^−1^ K^−1^ after reduction (A → F) and an increase from 1.8 to 5.9 W m^−1^ K^−1^ after oxidation (F → K). Both the reduction and oxidation processes exhibited a nearly linear change in *κ*. The slight variations in lattice constants between the oxidized and reduced states are attributed to the disappearance and generation of oxygen vacancies. Through phonon scattering, oxygen vacancies contribute to a decrease in the lattice thermal conductivity (*κ*_lat_).

However, there is a significant contrast in electrical conductivity (*σ*) between the oxidized (4250 S cm^−1^) and reduced (9 S cm^−1^) states (Table S4, Supporting Information). We estimated the *κ* *κ*_ele_ by assuming the Wiedemann–Franz law; *κ*_ele_ = *L*·*σ*·*T*, where *L* is the Lorentz number (2.44 × 10^−8^ W Ω K^−2^), and *T* is the absolute temperature. The *κ*_ele_ of the oxidized state reached 3.1 W m^−1^ K^−1^. According to the principle that observable *κ* is the sum of *κ*_lat_ and *κ*_ele_,^[^ ^22^ ^]^ we estimated the *κ*_lat_ of LaNiO_3_ (on‐state) to be 2.9 W m^−1^ K^−1^. This value aligns with previously reported values for bulk LaNiO_3_,^[^ ^20^ ^]^ confirming the consistency of our findings. This substantial difference enabled the LaNiO*_x_* thermal switch to modulate its *κ* over a wide range. Furthermore, the linear and reversible changes in *κ* indicate the potential of the LaNiO*_x_*‐based thermal switch for precise thermal modulation.

### 2.3 Cycle Properties of Thermal Switch

**Figure 5:**
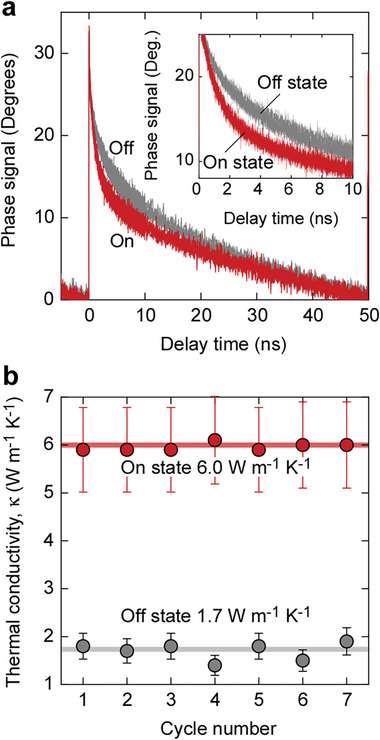
Cycle properties of LaNiO*_x_*‐based thermal switch. a) Changes in TDTR decay curves (seven cycles overlapped). The TDTR decay of the LaNiO_3_ layer was faster than that of the LaNiO_2.72_ layer. b) Change in *κ* of LaNiO_3_ and LaNiO_2.72_ layers after redox cycling. The average *κ* of the LaNiO_3_ (on‐state) layer and LaNiO_2.72_ (off‐state) layer were 6.0 and 1.7 W m^−1^ K^−1^, respectively. The *κ*_ele_ of the LaNiO_3_ (on‐state) layer was high (3.1 W m^−1^ K^−1^). The *κ*_ele_ of the LaNiO_2.72_ (off‐state) layer was negligible.

As shown in **Figures** **5** and S4 (Supporting Information), the LaNiO*_x_*‐based thermal switch exhibits cycling properties. The on‐state (LaNiO_3_) has a higher average *κ* (6.0 W m^−1^ K^−1^) than the off‐state (LaNiO_2.72_, 1.7 W m^−1^ K^−1^). Seven cycles are shown in the figure. The TDTR decay of LaNiO_3_ was faster than that of LaNiO_2.72_ (Figure 5a). The TDTR curves for each cycle overlapped significantly, indicating good repeatability.

### 2.4 Comparison with Other TMO Thermal Switches

**Figure 6:**
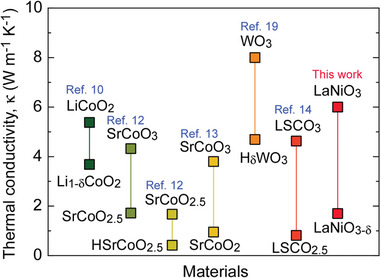
Comparison of *κ* switching widths of several transition metal oxides. LaNiO*_x_*‐based thermal switches exhibited large *κ* switching widths (≈4.3 W m^−1^ K^−1^). Data for LiCoO_2_ ↔ Li_1−_ *_δ_* CoO_2_ are from Ref. [10] SrCoO_3_ ↔ SrCoO_2.5_ and SrCoO_2.5_ ↔ HSrCoO_2.5_ are from Ref. [12] SrCoO_3_ ↔ SrCoO_2_ are from Ref. [13] WO_3_ ↔ H*_δ_* WO_3_ are from Ref. [19] and La_0.5_Sr_0.5_CoO_3_ (LSCO_3_) ↔ La_0.5_Sr_0.5_CoO_2.5_ (LSCO_2.5_) are from Ref. [14].

**Figure** **6** compares the *κ* switching widths of different TMOs, indicating the unparalleled performance of LaNiO*_x_*‐based thermal switches, with a *κ* switching width of ≈4.3 W m^−1^ K^−1^, greater than that of other TMOs. The wide switching range indicates the exceptional versatility of LaNiO*_x_* in modulating its *κ*. One more important property of thermal switches is their on‐to‐off *κ* ratio. Currently, La_0.5_Sr_0.5_CoO*_x_* (2.5 ≦ *x* ≦ 3) ^[^ ^14^ ^]^ based thermal switches show the largest on‐to‐off *κ* ratio of ≈5.4. It is still required to reduce the off‐state *κ* of LaNiO_3−_ *_δ_* to improve the on‐to‐off *κ* ratio (≈3.5). We will tackle this issue in future. The inherent ability of LaNiO*_x_*‐based thermal switches to linearly transition between high *κ* in the fully oxidized state and low *κ* in the reduced state positions them at good candidate TMOs for thermal management applications. The nuanced control over *κ* and the stability demonstrated in the cycling properties (Figure 5) underscore the potential of LaNiO*_x_*‐based thermal switches for application in next‐generation thermal displays and other advanced thermal management systems.

## 3 Conclusion

This study presents a breakthrough with LaNiO*_x_*‐based electrochemical thermal switches, with an exceptional *κ* switching width of 4.3 W m^−1^ K^−1^. The fully oxidized LaNiO_3_ (on‐state) produced a *κ* of 6.0 W m^−1^ K^−1^, primarily attributed to a substantial contribution from *κ*_ele_ (3.1 W m^−1^ K^−1^). In contrast, the reduced LaNiO_2.72_ (off‐state) had a low *κ* of 1.7 W m^−1^ K^−1^ due to negligible *κ*_ele_ (0.007 W m^−1^ K^−1^) and phonon scattering caused by oxygen vacancies. The LaNiO*_x_*‐based electrochemical thermal switch demonstrated *κ* cyclability while maintaining the structural integrity of the LaNiO*_x_* crystalline lattice, making it a promising candidate for integration into next‐generation devices, particularly thermal displays.

## 4 Experimental Section

### Fabrication of Thermal Switches

LaNiO_3_ films were heteroepitaxially grown on SrCoO*_x_*/10%‐Gd‐doped CeO_2_ (GDC)‐buffered (001)‐oriented YSZ substrates using pulsed laser deposition (PLD). First, ≈10 nm‐thick GDC was heteroepitaxially grown on a YSZ (10 mm × 10 mm × 0.5 mm, double‐sided polished, crystal base) substrate at 770 °C in an oxygen atmosphere (10 Pa). Approximately 2‐nm‐thick SrCoO*_x_* was grown on GDC in the same conditions. Focused KrF excimer laser pulses (*λ* = 248 nm, fluence ≈2 J cm^−2^ pulse^−1^, repetition rate = 10 Hz) were irradiated onto the ceramic target of GDC. Subsequently, an ≈80‐nm‐thick LaNiO_3_ film was heteroepitaxially grown on the GDC film at 625 °C in an oxygen atmosphere (25 Pa). The laser fluence was ≈1.6 J cm^−2^ pulse^−1^. After film growth, the sample was cooled to room temperature in a PLD chamber in an oxygen atmosphere (25 Pa). An ≈50‐nm‐thick Pt film was sputtered on the top surface of the LaNiO_3_ epitaxial film, followed by an ≈50‐nm‐thick Pt film sputtered on the backside of the YSZ substrate. DC sputtering of Pt was performed at room temperature. The samples were cut into four squares (≈5 mm × 5 mm).

### Electrochemical Redox Treatment

Thermal switch (≈5 mm × 5 mm) was placed on a Pt‐coated glass substrate and heated at 280 °C in air. Electrochemical redox treatment was performed by applying a constant current of ±10 µA, after which the sample was immediately cooled to room temperature. During the redox treatment, it controlled the flown electron density *Q* = (*I·t*)/(*e·V*) through the current application time.

### Crystallographic Analyses

The crystalline phase, orientation, and lattice parameters of the resultant films were analyzed using high‐resolution X‐ray diffraction (Cu Kα_1_, *λ* = 1.54059 Å, ATX‐G, Rigaku). Out‐of‐plane Bragg diffraction patterns and reciprocal space mappings (RSMs) were measured at room temperature to clarify changes in the crystalline phase of LaNiO*_x_*. The lattice parameters were calculated from the diffraction peaks. The atomic arrangements of the LaNiO_3_ films were visualized using a STEM (JEM‐ARM200CF, JEOL) operated at 200 keV.

### Measurement of Electrical Properties of LaNiO_x_ Layers

To measure the electrical conductivity (*σ*) of the LaNiO*_x_* layers after redox treatment, it mechanically attached Au foil on the film surface while Pt films were deposited only on the backside of the YSZ substrate.^[^ ^13^ ^]^ The LaNiO*_x_* films were oxidized and reduced electrochemically at 280 °C in air using the Au foil as the electrode. The *σ* of the LaNiO_3_ (on‐state) and LaNiO_2.72_ (off‐state) films was measured using the DC four‐probe method with a van der Pauw electrode configuration at room temperature in air.

### Thermal Conductivity Measurements

The *κ* of the LaNiO_*x*_ layers perpendicular to the substrate surface was measured through time‐domain thermoreflectance (TDTR, PicoTR, Netzsch Japan). The top Pt film was used as the transducer. The decay curves of the TDTR signals were simulated to obtain *κ*. The specific heat capacity of the LaNiO_3_ ceramic target was measured by differential scanning calorimetry (DSC, DSC200, Hitachi High‐Tech Co.). The specific heat capacities of the layers used for the TDTR simulation were Pt: 132 J kg^−1^ K^−1[^ ^23^ ^]^; LaNiO_3_: 420 J kg^−1^ K^−1^ (measured) (*cf*. 448 J kg^−1^ K^−1[^ ^24^ ^]^), and YSZ: 460 J kg^−1^ K^−1[^ ^25^ ^]^. Details of the TDTR method are described in the previous studies.^[^ ^13^, ^17^, ^26^, ^27^, ^28^ ^]^ Note that it simulated the TDTR decay curves by assuming the interfacial resistance due to 2‐nm‐thick SrCoO*_x_* and 10‐nm‐thick GDC layers is constant (1 × 10^−9^ K W^−1^). It confirmed this assumption works by measuring the *κ* of the thicker (151‐nm‐thick) LaNiO_3_ active layer. In the treatment of the *κ* values, as there were uncertainties such as the position of the baseline, position of time zero, and noise in the signal, it used error bars for ±15% of the obtained values.

## Conflict of Interest

The authors declare no conflict of interest.

## Supporting information

Supporting Information

## Data Availability

The data that support the findings of this study are available in the supplementary material of this article.
