# Supplementary material for: Solid‐State Electrochemical Thermal Switches with Large Thermal Conductivity Switching Widths
Source: Adv Sci (Weinh). 2024 Jun 25;11(32):2401331. doi: 10.1002/advs.202401331 (PMC11348128; doi:10.1002/advs.202401331)
Supplement: Supplementary file 1 — Supporting Information [file ADVS-11-2401331-s001.docx]

Supporting Information

Solid-State Electrochemical Thermal Switches with Large Thermal Conductivity Switching Widths

Zhiping Bian, Mitsuki Yoshimura, Ahrong Jeong, Haobo Li, Takashi Endo, Yasutaka Matsuo, Yusaku Magari, Hidekazu Tanaka, Hiromichi Ohta*

S1: Selection of LaNiO_3_ as the active layer (Table S1, Table S2, Figure S1)

S2. Fabrication of LaNiO_3_-based solid-state thermal switches (Figure S2, Table S3, Table S4)

S3. Repeated thermal conductivity measurements of LaNiO*_x_*-based solid-state thermal switches (Figure S3, Figure S4)

**S1. Selection of LaNiO_3_ as the active layer**

In this study, we focused on perovskite-structured *Ln*NiO_3_ (*Ln* = La, Nd, and Sm) as the active layer for the solid-state electrochemical thermal switch. **Table S1** summarizes the electrical conductivity (*σ*) and the thermal conductivity (*κ*) of bulk *Ln*NiO_3_ (*Ln* = La, Nd, and Sm) at room temperature^[1]^. At room temperature, bulk *Ln*NiO_3_ (*Ln* = La, Nd, and Sm) shows the following *κ*; LaNiO_3_: 10.7 W m^−1^ K^−1^, NdNiO_3_: 6.5 W m^−1^ K^−1^, and SmNiO_3_: 4.0 W m^−1^ K^−1^. Thus, LaNiO_3_ is a promising candidate as the active layer of the thermal switches. It should be noted that the *σ* of LaNiO_3_ is 10500 S cm^−1^. We assumed the Wiedemann-Frantz law for the estimation of electron contribution to the observed thermal conductivity (*κ*_ele_ = *L*·*σ*·*T*, where *L* is the Lorentz number of 2.44 × 10^−8^ W Ω K^−2^ and *T* is the absolute temperature of 298 K) and obtained *κ*_ele_ of 7.6 W m^−1^ K^−1^ for LaNiO_3_. This reflects the lattice thermal conductivity (*κ*_lat_) of LaNiO_3_ is 3.1 W m^−1^ K^−1^.

**Table S1**. **The electrical conductivity (*σ*) and the thermal conductivity (*κ*) of bulk *Ln*NiO_3_ (*Ln* = La, Nd, and Sm) at room temperature.** The ionic radius data is from Shannon’s report.^[2]^

| J.-S. Zhou *et al*., *PRB* **67**, 020404(R) (2003)^[1]^ | LaNiO_3_ | NdNiO_3_ | SmNiO_3_ |
| --- | --- | --- | --- |
| Ionic radius of *Ln*^3+^ ion (Å) (C.N. = 12) | 1.36 | 1.27 | 1.24 |
| Electrical conductivity at RT (S cm^−1^) | 10500 | 3400 | --- |
| Total thermal conductivity, *κ* (W m^−1^ K^−1^) | 10.7 | 6.5 | 4.0 |
| Electron thermal conductivity, *κ*_ele_ (W m^−1^ K^−1^) | 7.6 | 2.5 | 0 |
| Lattice thermal conductivity, *κ*_lat_ (W m^−1^ K^−1^) | 3.1 | 4.0 | 4.0 |

To check the potential of LaNiO_3_ epitaxial films as the active layer of the thermal switches, we fabricated LaNiO_3_ epitaxial films on (001) SrTiO_3_ substrates and measured the electrical and thermal conductivity of the resultant films at room temperature (**Table S2**). The *σ* of the resultant LaNiO_3_ film was only 135 S cm^−1^, two orders of magnitude smaller than that of bulk. The *κ* in the out-of-plane of the LaNiO_3_ film was 7.3 W m^−1^ K^−1^. If we assumed the Wiedemann-Frantz law for the estimation of *κ*_ele_, the *κ*_ele_ of the LaNiO_3_ film was only 0.1 W m^−1^ K^−1^, reflecting the *κ*_lat_ of LaNiO_3_ is 7.2 W m^−1^ K^−1^.

**Table S2**. **The electrical and thermal conductivity of the LaNiO_3_ epitaxial films on (001) SrTiO_3_ at room temperature.**

| This study | LaNiO_3_ |
| --- | --- |
| Ionic radius of *Ln*^3+^ ion (Å) (C.N. = 12) | 1.36 |
| Electrical conductivity at RT (S cm^−1^) | 135 |
| Total thermal conductivity, *κ* (W m^−1^ K^−1^) | 7.3 |
| Electron thermal conductivity, *κ*_ele_ (W m^−1^ K^−1^) | 0.1 |
| Lattice thermal conductivity, *κ*_lat_ (W m^−1^ K^−1^) | 7.2 |

Since the estimated *κ*_lat_ of the LaNiO_3_ film on (001) SrTiO_3_ substrate is higher than that of the bulk, there is a possibility that we underestimated the *κ*_ele_ of the LaNiO_3_ film. To clarify the origin of it, we observed the microstructure using Cs-corrected scanning transmission electron microscopy (**Fig. S1**). The columnar structure is visualized in the LaNiO_3_ film (**Fig. S1a**). The magnified image (**Fig. S1b**) reveals that there are many planer defects due to the formation of Ruddlesden-Popper phases. Since the electron transport is suppressed by the planer defects, the observed electrical conductivity in the in-plane direction would be lower than that in the out-of-plane direction.


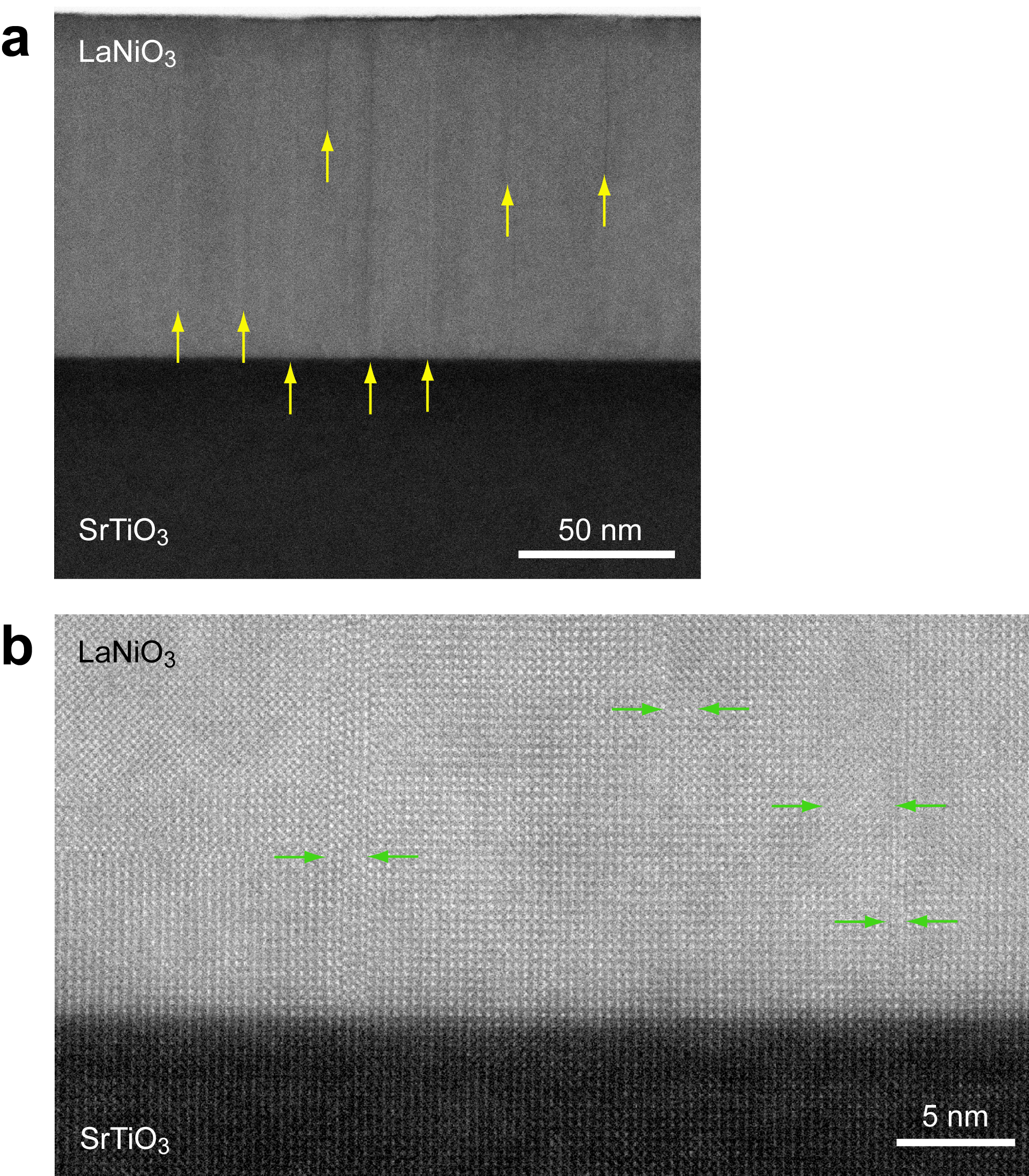


**Figure S1. Microstructure of the LaNiO_3_ film grown on (001) SrTiO_3_ substrate.** (a) Low magnification cross-sectional HAADF-STEM image. Stripe patterns (yellow arrows) indicate that columnar growth of LaNiO_3_ occurred. (b) Lattice image around the LaNiO_3_/SrTO_3_ heterointerface. Planar defects (green arrows) are visualized.

**S2. Fabrication of LaNiO_3_-based solid-state thermal switches**

Firstly, we fabricated LaNiO_3_ films directly on (001) YSZ substrates. **Figure S2a** shows the out-of-plane XRD pattern of the resultant film. Together with 001 and 002 diffraction peaks of LaNiO_3_, 110 diffraction peaks of LaNiO_3_ are seen with 002 YSZ, indicating the mixed orientation of the film. The out-of-plane rocking curve of the 002 LaNiO_3_ (**Fig. S2d**) is broad (the full width at half maximum, FWHM ~3.1°). Then, we fabricated LaNiO_3_ films on GDC-buffered (001) YSZ substrates. As shown in **Fig. S2b**, intense diffraction peaks of 001 and 002 LaNiO_3_ are seen together with 002 GDC and 002 YSZ. The FWHM of the 002 LaNiO_3_ is 1.6° as shown in **Fig. 2e**. The reciprocal space mapping (RSM, not shown) of the film revealed that the LaNiO_3_ is heteroepitaxially grown on the GDC-buffered YSZ substrate. To further improve the crystallographic orientation of the film, we inserted thin (~2 nm) SrCoO*_x_* layer between the LaNiO_3_ and GDC-buffered substrate. The out-of-plane XRD peaks became stronger (**Fig. S2c**) and the tilting became small (1.2°) as shown in **Fig. S2f**.


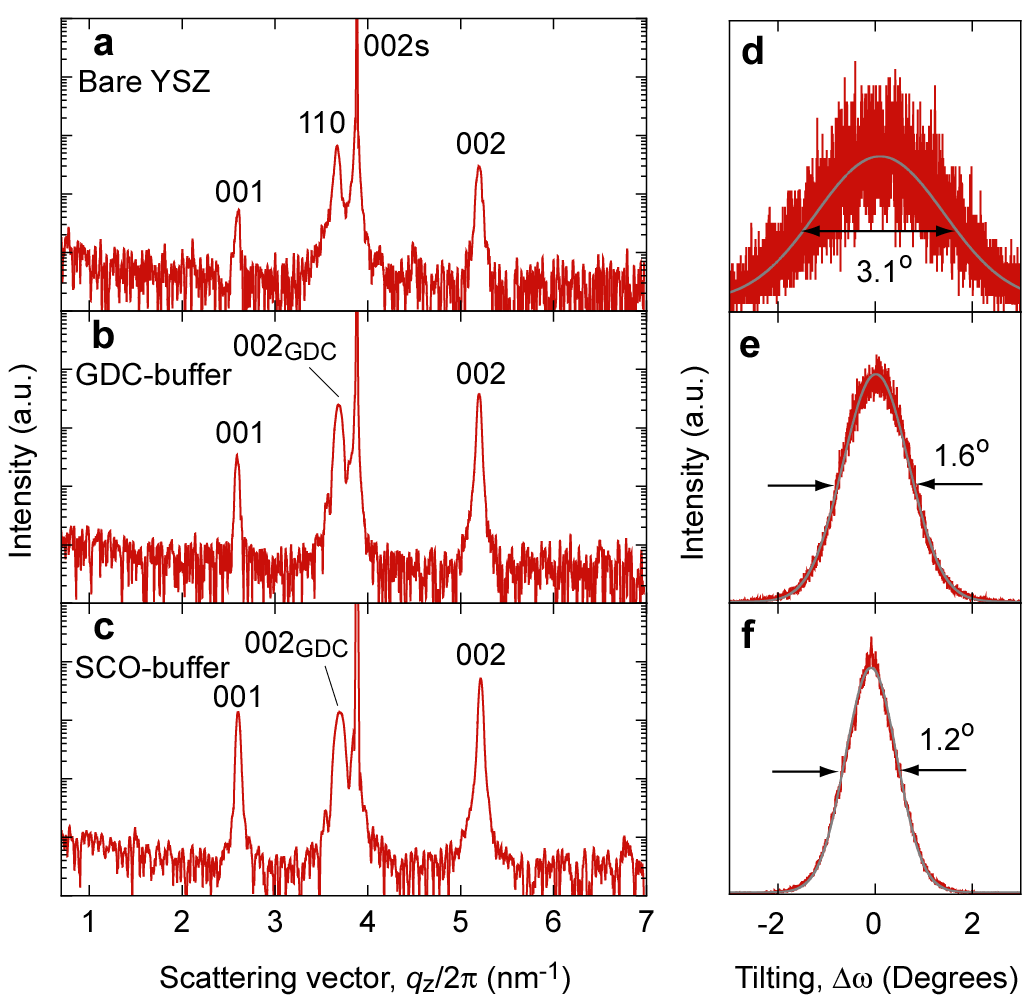


**Figure S2. XRD patterns of the LaNiO_3_ films grown on various substrates.** (a, b, c) Out-of-plane Bragg diffraction patterns of the LaNiO_3_ films grown on (a) bare (001) YSZ substrate, (b) GDC-buffered (001) YSZ substrate, and (c) SrCoO*_x_*/GDC-buffered (001) YSZ substrate. (d, e, f) Out-of-plane rocking curves of 002 LaNiO_3_ of the films on (d) bare (001) YSZ substrate, (e) GDC-buffered (001) YSZ substrate, and (f) SrCoO*_x_*/GDC-buffered (001) YSZ substrate.

Then, we measured the *σ* and the *κ* of the resultant LaNiO_3_ films on the various substrates at room temperature. **Table S3** summarizes the results. The LaNiO_3_ film on the SrCoO*_x_*-buffered substrate showed the highest *σ* of 4200 S cm^−1^, reflecting the improvement of the crystallographic orientation of the LaNiO_3_ film. The out-of-plane *κ* of the LaNiO_3_ film on the SrCoO*_x_*-buffered substrate was the highest (5.7 W m^−1^ K^−1^) among the films on different three substrates. We estimated the *κ*_ele_ and the *κ*_lat_ of the LaNiO_3_ films. The *κ*_lat_ of the LaNiO_3_ film on SrCoO*_x_*-buffered substrate was 2.7 W m^−1^ K^−1^, similar to that of bulk (3.1 W m^−1^ K^−1^).

**Table S3**. **Electrical and thermal conductivity of the LaNiO_3_ films grown on the three different substrates.**

|  | Bare YSZ | GDC-buffered | SCO-buffered |
| --- | --- | --- | --- |
| Electrical conductivity, *σ* (S cm^−1^) | 500 | 1200 | 4200 |
| Thermal conductivity, *κ* (W m^−1^ K^−1^) | 1.6 | 4 | 5.7 |
| Electron thermal conductivity, *κ*_ele_ (W m^−1^ K^−1^) | 0.36 | 0.88 | 3.0 |
| Lattice thermal conductivity, *κ*_lat_ (W m^−1^ K^−1^) | 1.2 | 3.1 | 2.7 |

Then, we reduced the LaNiO_3_ film on SCO-buffered (001) YSZ substrate electrochemically, and measured the *σ* and the *κ* (**Table S4**). The reduction treatment by applying total *Q* of 1 × 10^22^ cm^−3^ results in the significant reduction of both *σ* and the *κ*.

**Table S4. Electrical and thermal conductivity of the LaNiO_3_ films on SrCoO*_x_*/GDC-buffered substrate (oxidized state and reduced state).**

|  | Oxidized | Reduced |
| --- | --- | --- |
| Electrical conductivity, *σ* (S cm^−1^) | 4200 | 9 |
| Thermal conductivity, *κ* (W m^−1^ K^−1^) | 5.9 | 1.8 |
| Electron thermal conductivity, *κ*_ele_ (W m^−1^ K^−1^) | 3.1 | 0.006 |
| Lattice thermal conductivity, *κ*_lat_ (W m^−1^ K^−1^) | 2.8 | 1.8 |

**S3. Repeated thermal conductivity measurements of the LaNiO_3_-based solid-state thermal switches**


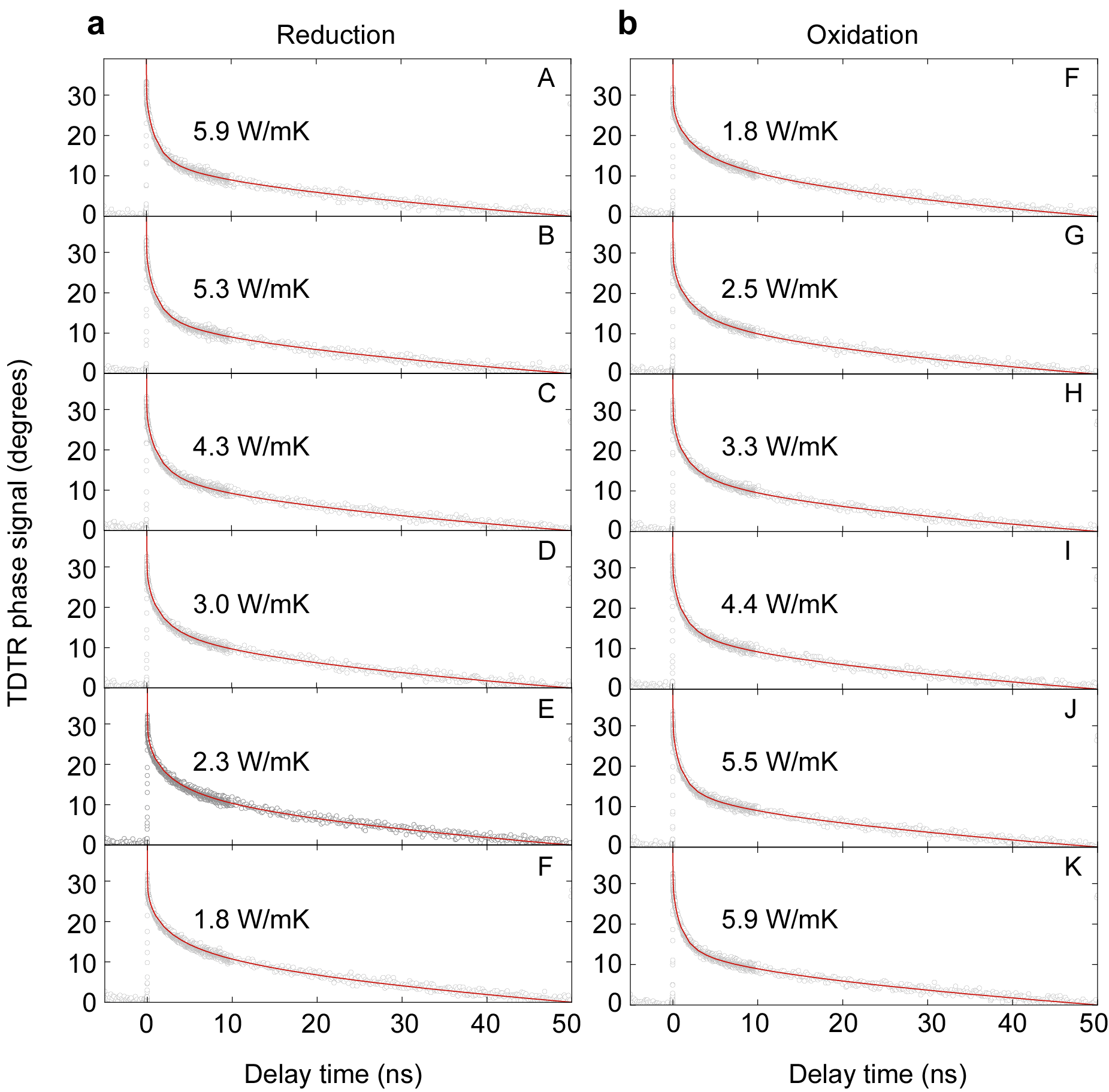


**Figure S3. Change in the thermal conductivity of the LaNiO_3_ layer in the thermal switch.** (a, b) TDTR phase signal decay curves during (a) reduction and (b) oxidation. The reduction treatment was performed in the order of A, B, C,…F with a step of *Q* = 2 × 10^21^ cm^−3^. The oxidation treatment was performed in the order of F, G, H,…K with a step of *Q* = 2 × 10^21^ cm^−3^.


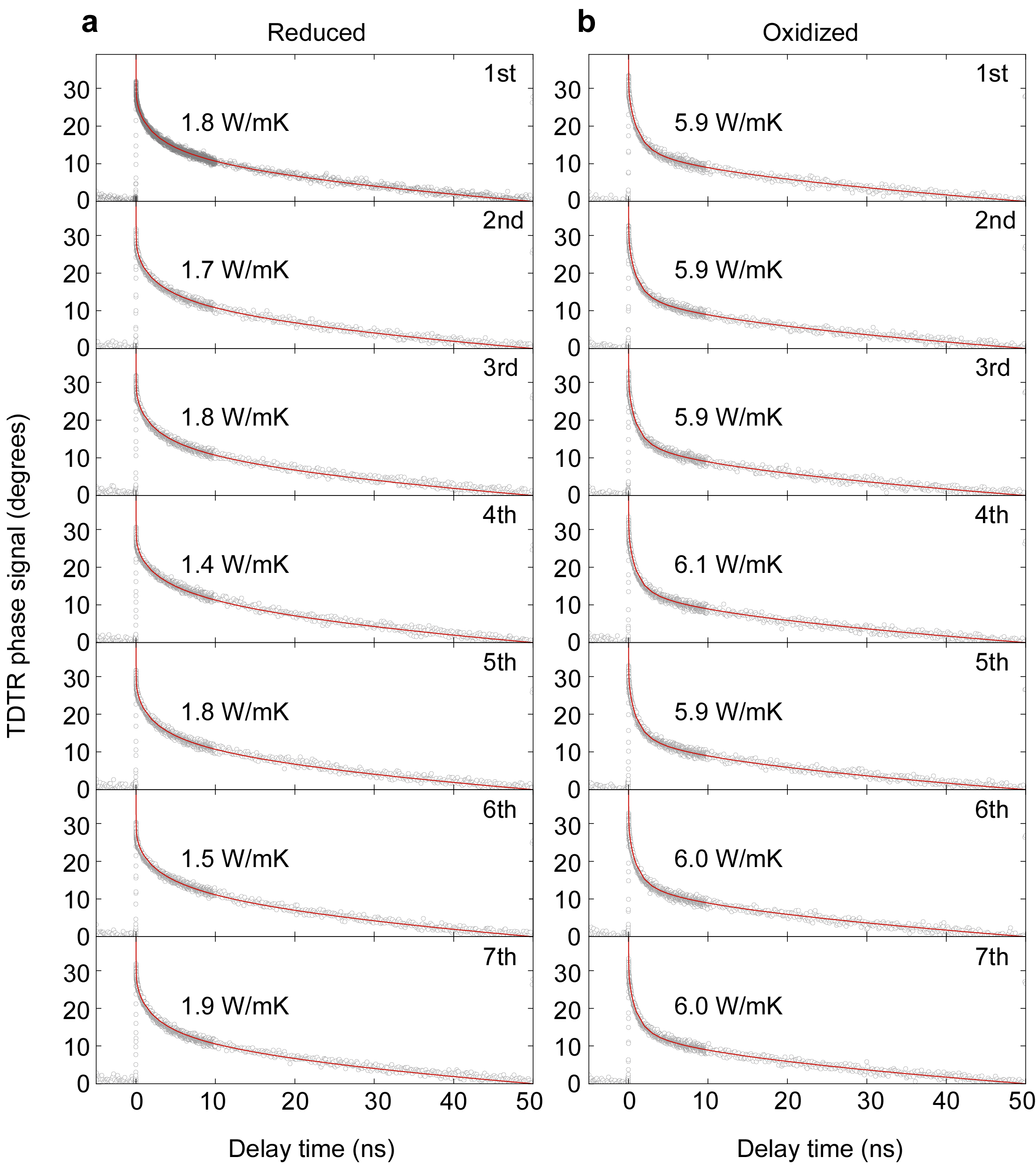


**Figure S4. TDTR decay cycle of the LaNiO_3_ layer in the thermal switch.** (a, b) TDTR phase signal decay curves after (a) reduction and (b) oxidation.

**References**

[1] J. S. Zhou, J. B. Goodenough, B. Dabrowski, *Phys. Rev. B* **2003**, *67*, 020404 (R).

[2] R. D. Shannon, *Acta Crystallogr. Sec. A* **1976**, 32, 751-767.
